# Detection of the Virulent Form of AVR3a from *Phytophthora infestans* following Artificial Evolution of Potato Resistance Gene *R3a*

**DOI:** 10.1371/journal.pone.0110158

**Published:** 2014-10-23

**Authors:** Sean Chapman, Laura J. Stevens, Petra C. Boevink, Stefan Engelhardt, Colin J. Alexander, Brian Harrower, Nicolas Champouret, Kara McGeachy, Pauline S. M. Van Weymers, Xinwei Chen, Paul R. J. Birch, Ingo Hein

**Affiliations:** 1 Cell and Molecular Sciences, James Hutton Institute, Invergowrie-Dundee, United Kingdom; 2 Division of Plant Sciences, University of Dundee at James Hutton Institute, Invergowrie-Dundee, United Kingdom; 3 Dundee Effector Consortium, Invergowrie-Dundee, United Kingdom; 4 Biomathematics and Statistics Scotland, Invergowrie-Dundee, United Kingdom; 5 J.R. Simplot Company, Simplot Plant Sciences, Boise, Idaho, United States of America; Leibniz-Institute for Vegetable and Ornamental Crops, Germany

## Abstract

Engineering resistance genes to gain effector recognition is emerging as an important step in attaining broad, durable resistance. We engineered potato resistance gene *R3a* to gain recognition of the virulent AVR3a^EM^ effector form of *Phytophthora infestans*. Random mutagenesis, gene shuffling and site-directed mutagenesis of *R3a* were conducted to produce R3a* variants with gain of recognition towards AVR3a^EM^. Programmed cell death following gain of recognition was enhanced in iterative rounds of artificial evolution and neared levels observed for recognition of AVR3a^KI^ by R3a. We demonstrated that R3a*-mediated recognition responses, like for R3a, are dependent on SGT1 and HSP90. In addition, this gain of response is associated with re-localisation of R3a* variants from the cytoplasm to late endosomes when co-expressed with either AVR3a^KI^ or AVR3a^EM^ a mechanism that was previously only seen for R3a upon co-infiltration with AVR3a^KI^. Similarly, AVR3a^EM^ specifically re-localised to the same vesicles upon recognition by R3a* variants, but not with R3a. R3a and R3a* provide resistance to *P. infestans* isolates expressing AVR3a^KI^ but not those homozygous for AVR3a^EM^.

## Introduction

In a process known as effector triggered immunity (ETI), plant disease resistance (*R*) genes can facilitate immunity to phylogenetically diverse and unrelated pathogens that express cognate effector molecules [1]. Effectors that are recognised by *R* genes, either directly or indirectly, and provoke successful plant defences are genetically defined as avirulence (*Avr*) genes. The best described group of plant *R* gene products contains a nucleotide-binding (NB) domain and leucine-rich repeats (LRRs), collectively known as NB-LRRs [2]. NB-LRRs are strictly regulated by plants as, upon their activation, many elicit programmed cell death (PCD) as part of the hypersensitive response (HR) which prevents further spread of disease in plant tissues [3]. Together with effectors, *R* genes are at the forefront of host/pathogen co-evolution [4]. NB-LRRs are one of the largest gene families in plants and more than 750 members have recently been described in potato [5]–[6]. The organisation of many NB-LRRs into physically-linked clusters is providing insight into their evolution, which can involve duplication followed by diversification.

In agriculture, successful deployment of *R* genes to control important diseases in crop plants has so far been hampered by the ability of pathogens often to rapidly circumvent detection by the host plant's innate immune system. Advances in studying pathogen effector diversity coupled with the ability to engineer *R* genes offers the opportunity to develop more durable resistances that specifically target essential effectors and known variants [7]–[8].

The *Phytophthora infestans* effector AVR3a is an essential effector for this pathogen to cause late blight on potato. Stable silencing of *Avr3a* in the *P. infestans* isolate 88069 significantly reduces infection in susceptible *Solanum tuberosum* (potato) cv. Bintje and in the model solanaceous plant species *Nicotiana benthamiana* [9]–[10]. Two forms of AVR3a are prevalent in current *P. infestans* isolates and differ in only two amino acids within the mature protein; AVR3aE^80^M^103^ (AVR3a^EM^) and AVR3aK^80^I^103^ (AVR3a^KI^) [11]–[12]. AVR3a^KI^ elicits ETI upon recognition by the potato resistance protein R3a, a member of the coiled-coil (CC) NB-LRR gene family [13]. This response is evaded by AVR3a^EM^ which consequently is free to promote virulence [9], [11], [13]. However, a weak R3a-dependent response to AVR3a^EM^ can be observed under UV light [14]. The mechanism of R3a-mediated recognition of AVR3a has been investigated recently [15]. Upon activation by AVR3a^KI^, both the effector protein and R3a rapidly re-localize from the host cytoplasm to late endosomes, components of the endocytic pathway, which is thought to be a prerequisite for subsequent HR development. The un-recognised AVR3a^EM^ form of the effector does not cause this re-localisation. There is no evidence of direct interaction between AVR3a^KI^ and R3a, but bimolecular fluorescence complementation (BiFC) assays reveal that the two proteins are in close proximity at late endosomes [15].

Artificial evolution has previously been used to alter the recognition specificity of the potato CC-NB-LRR resistance protein Rx to gain recognition of different strains of *Potato virus X* (PVX) and a distantly related virus, *Poplar mosaic virus* (PopMV) [16]–[17]. Random mutagenesis, screening for beneficial mutations and designed amalgamation of these mutations has been used to generate transgenic plants of the model species *N. benthamiana* that are resistant to previously virulent strains. DNA shuffling, also known as directed evolution, was first developed in the early 1990 s and has since been used to generate a wide variety of novel genes and proteins [18]. DNA shuffling has previously been used in the functional analysis of the resistance gene *Pto* [19]. In this study, the LRR of *R3a* has been subjected to error-prone PCR and iterative rounds of DNA shuffling to identify gain-of-recognition variants (R3a*) through functional screening in *N. benthamiana*. R3a* gene products with varying degrees of AVR3a^EM^ recognition were generated in three rounds of DNA shuffling and a subsequent round of site-directed mutagenesis. The best-performing clones from each round were taken forward for further analysis and compared to wild-type R3a. R3a* variants demonstrated significantly improved gain-of-recognition towards AVR3a^EM^ that manifested itself as a gain of re-localisation to late endosomes, but not yet as a gain of resistance.

## Materials and Methods

### Construction of plasmid vectors

The plasmid pBinPlus.R3a [13] was amplified with the primer pairs R3a-5-Asc/R3a-1564-Bam-M and R3a-1564-Bam-P/R3a-3-Not (Table S1) and the *Asc*I/*Bam*HI and *Bam*HI/*Not*I digested amplification products cloned in a three way ligation in to *Asc*I/*Not*I digested binary vector pGRAB [20] to produce pGRAB.35S::R3a. A derivative of the former plasmid, pGRAB.R3a::R3a, was produced in which the *Cauliflower mosaic virus* 35S (35S) promoter sequence was replaced with the *R3a* promoter sequence. This derivative was produced by digestion of pBinPlus.R3a with *Pme*I and *Xho*I, and insertion of the released fragment, containing 2358 bases upstream of the translational start site, in to pGRAB.35S::R3a that had been treated with *Bsp*EI, T4 DNA polymerase and *Xho*I in order.

### Mutagenesis and DNA shuffling

Shuffling of 2283 bp of the R3a LRR region was performed as described by Stemmer [18]. First round PCRs were carried out with the primer pair R3a-1564-Bam-P/R3a-3-Not in the presence of 0.5 mM MnCl_2_ as described by Leung *et al.* [21] with *Taq* DNA polymerase (Roche, Mannheim, Germany). Following DNaseI treatment, fragments of circa 500 bp were reassembled through forty rounds of primer-less thermo-cycling. Gel-purified products of circa 2.3 kb were amplified through thirty cycles of PCR and, following gel-purification, digested with *Bam*HI and *Not*I prior to cloning in to pGRAB.35S::R3a digested with the same enzymes. The ligated population was amplified in *E. coli* resulting in a population with a complexity of circa 125 K, a vector background of circa 14% and a base mutation rate of 0.43%. Aliquots of plasmid, gel-purified on account of plasmid instability, were transformed in to *A. tumefaciens* cells. In the second and third rounds *PfuUltra* II Fusion HS DNA polymerase (Stratagene, La Jolla, CA, USA) was used in thermo-cycling reactions to produce shuffled populations with lower mutation rates, less than 0.05%. The second and third round populations had complexities of circa 200 K and 150 K, respectively, and both had a vector background of circa 10%.

### Site-directed mutagenesis

A mixture of two templates, the wild-type gene and clone Rd1-2 from the first round of shuffling containing the Q931R codon substitution, was used in PCR amplifications with the primer pairs R3a-1564-Bam-P/R3a-1740W-M, R3a-1740W-P/R3a-1841W-M, R3a-1841W-P/R3a-2743W-M, R3a-2743W-P/R3a-3028-M. The products of the primary PCRs were used in an overlap PCR reaction with the flanking primer pair R3a-1564-Bam-P/R3a-3028-M. The product of the secondary PCR reaction was digested with *Bam*HI and *Aat*II and cloned between the same unique sites of pGRAB.R3a::R3a. A population of circa 50K clones, with a vector background of 1% and random base mutation rate of 0.05% was produced.

### Plant growth conditions

*N. benthamiana* plants were grown in a glasshouse with a 16 h day period at 22°C and an 8 h night period at 18°C. Supplementary lighting was provided below 200 W m^−2^ and screening above 450 W m^−2^.

### Screening of mutated R3a clones

DNA populations prepared from *E. coli* were transformed in to *Agrobacterium tumefaciens* strain AGL1 [22] carrying the helper plasmids pSoup and pBBR1MCS1.VirG_N54D_ [23] for screening. *Agrobacterium* cultures grown from single transformed colonies were co-infiltrated with cultures of *Agrobacterium* transformed with pGRAB.35S::AVR3a^EM^ according to the method of Engelhardt *et al.* [15] in to *N. benthamiana* leaves with each of the components at the same final OD_600_ of 0.1–0.5. Reference mixtures were infiltrated in to opposing half-leaves and between two and seven days post infiltration leaves were inspected to assess visible symptoms and plant auto-fluorescence under day-light and 365 nm illumination from a Blak-Ray lamp (UVP, Upland, CA, USA), respectively.

### Symptom scoring

Circa five week old plants were used for symptom scoring with two adjacent, expanded leaves, both circa 90 mm in length, being used for infiltrations. Symptoms were scored on an arbitrary scale for nine days after infiltration. Symptom scores were plotted; the areas under the curve determined and mean scores calculated by dividing by the duration. A linear mixed modelling approach was adopted using GenStat for Windows, 16^th^ edition (VSN International Ltd., Hemel Hempstead, UK). The data were analysed in two stages: first, the stability of the phenotypes over repeated experiments was examined by fitting a model with experiment as a fixed effect. Infiltration mixture, leaf age, position of infiltration site on leaf, experiment and their interactions were set as fixed effects with plant and leaf within an individual plant as the random effects. This allowed for terms in the model which specifically tested for significant interactions with experiment. Lack of any significant interaction with the infiltration mixtures would provide evidence that the relative responses of the phenotypes were consistent. Having determined that the infiltration mixtures behaved consistently over the experiments, the second stage fitted a model with experiment as a random effect with plant and leaf within plant nested below this. Multiple comparison tests then examined the differences in response amongst the infiltration mixtures.

### Virus-induced gene silencing (VIGS) of *SGT1* and *HSP90*

*Tobacco rattle virus* (TRV)-induced gene silencing in *N. benthamiana* was performed as described previously [14]. *Agrobacterium* cultures transformed with the binary TRV RNA1 construct, pBINTRA6, or the TRV RNA2 vector constructs PTV00, PTV:*eGFP*, PTV:*HSP90* or PTV:*SGT1* were re-suspended to OD_600_  = 0.5 for the RNA1 construct and OD_600_  = 1.0 for the RNA2 constructs. Re-suspended RNA1 and RNA2 cultures were mixed in a 1∶1 ratio and infiltrated into non-cotyledonous leaves of *N. benthamiana* plants at the 5-leaf stage. For each of the biological replicates, six plants per treatment were used and six plants were used as non-TRV controls. Three weeks after treatment with the VIGS constructs, plants were infiltrated with culture mixtures (OD_600_  = 0.5) designed to express R3a, Rd2-1, Rd3-1 or Rd4-1 and AVR3a^KI^ or AVR3a^EM^. HRs were scored at 6dpi.

### Confocal laser scanning microscopy

Imaging was performed on a Leica TCS-SP2 AOBS microscope (Leica Microsystems) using HCX APO L, 40x/0.8, and 63x/0.9 water dipping lenses or a Zeiss 710 using a Plan APO 40x/1.0 water dipping lens. Images were collected using line by line sequential scanning. The optimal pinhole diameter and the same gain levels were used within experiments. YFP and CFP were imaged using 514 nm and 405 nm excitation, respectively, and emissions were collected between 520–563 nm and 455–490 nm, respectively. Photoshop CS5.1 software (Adobe Systems) was used for post-acquisition image processing.

### Agrobacterium tumefaciens transient assays (ATTAs)

Functional *Agrobacterium tumefaciens* transient assays (ATTAs) were carried out in *N. benthamiana*. Cultures carrying pGRAB.R3a::R3a, pGRAB.R3a::Rd2-1, pGRAB.R3a::Rd3-1, pGRAB.R3a::Rd4-1 or pGRAB empty vector were re-suspended as described before to OD600  = 0.1 for each construct. Each of the five resuspensions was infiltrated into separate areas of leaves. Four leaves on each of sixteen plants were infiltrated in each replicate. Two days post infiltration, leaves were detached and infiltration sites inoculated with *AVR3a^KI^* homozygous *P. infestans* isolate 7804.b or *AVR3a^EM^* homozygous isolate 88069. Leaves were incubated in transparent sealed boxes at 100% humidity in a cool room and covered for the first 12 hours. Lesion sizes were measured up to 15dpi.

### Production of transgenic potato plants

*R3a* wild-type gene and the three modified versions Rd2-1, Rd3-1 and Rd4-1 were cloned under *R3a* native regulatory elements in a pCambia-based binary vector with kanamycin resistance as a selectable marker, using standard restriction enzyme methods to create pSIM2093, pSIM3027, pSIM3028 and pSIM3029 respectively. *Rpi-vnt1* under its native regulatory elements was cloned in to pSIM401 to generate pSIM1620. The binary vectors were then transformed into *Agrobacterium* strains AGL1 and LBA4404 for plant transformation. Ranger Russet was transformed as described in Duan *et al*. [24]. For each construct twenty to thirty lines were regenerated with kanamycin selection. These were tested for late blight resistance and assessed at 7dpi.

## Results

### Identification of R3a mutants with enhanced AVR3a^EM^ recognition

**Figure 1:**
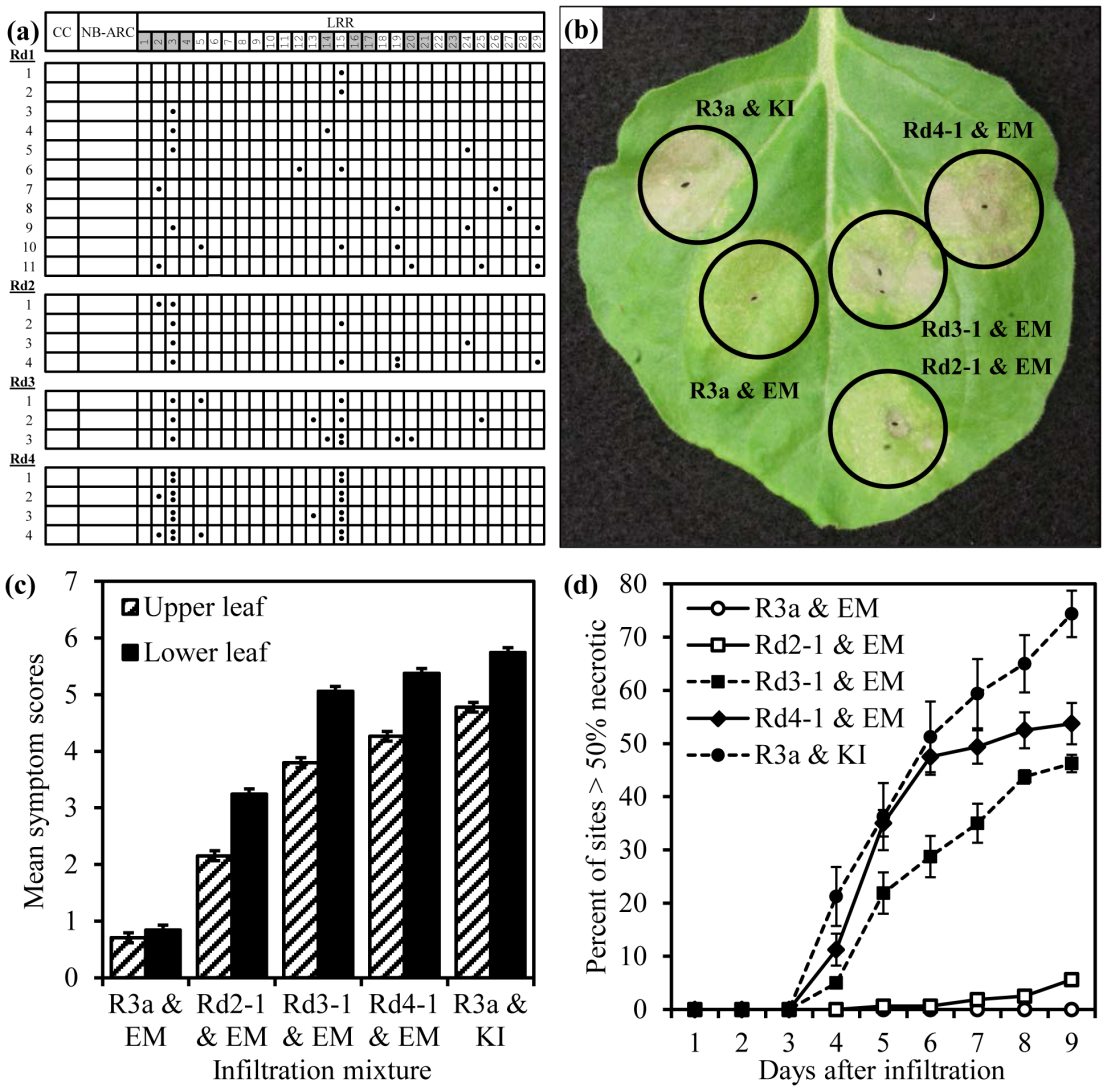
Four rounds of mutagenesis and shuffling identified R3a mutants with enhanced recognition of AVR3a^EM^ and disease responses (R3a*). (a) Schematic showing locations of non-synonymous mutations found in the LRRs of R3a* clones isolated from the four rounds of mutagenesis and shuffling (Rd1 to Rd4). LRRs containing amino acids under diversifying selection are shaded above. (b) Representative *N. benthamiana* leaf showing responses of best performing clones from second, third and fourth rounds (Rd2-1, Rd3-1 & Rd4-1) to AVR3a^EM^ (EM), compared to responses of wild-type R3a to AVR3a^KI^ (KI) and AVR3a^EM^ five days after co-infiltration with resistance genes under transcriptional control of *R3a* promoter. (c) Mean disease scores from the four experiments, each of nine days duration, for different infiltration mixtures in upper (hatched) and lower (solid) paired leaves. Error bars show +/− standard error. (d) Time-course of percentage of sites showing necrosis development, greater than 50% necrosis of individual infiltrated sites, for the five infiltrated mixtures. Mean percentages of the four experiments. Each experiment includes data for 40 infiltration sites (upper and lower leaves combined) and error bars show +/− standard errors.

A binary vector, pGRAB.35S::R3a, containing the R3a open reading frame (Accession number AY849382.1) under the control of *Cauliflower mosaic virus* 35S promoter and terminator sequences was produced to allow specific mutation and shuffling of the R3a LRR domain. In this plasmid a unique *Bam*HI site was introduced silently at nucleotide position 1567 in the ORF, ninety nucleotides upstream of the sequence encoding the LRR domain as denoted by Huang *et al.* [13]. Following mutagenesis and DNA shuffling of the LRR region, regenerated recombinant clones were screened for enhanced AVR3a^EM^ recognition through *Agrobacterium*-mediated transient expression in co-infiltrations with binaries expressing the virulent elicitor, AVR3a^EM^. Clones were screened for enhanced recognition through comparison of visible symptoms of PCD and induced auto-fluorescence with reference to the responses produced by the wild-type R3a gene and AVR3a^EM^. Clones with putatively improved recognition isolated from primary screens were screened again to confirm the phenotypic improvement and to eliminate auto-activators. In the first cycle, screening of approximately three thousand clones identified eleven R3a* clones with improved phenotypes. The eleven clones from the first round are referred to as Rd1-1 to Rd1-11 and contained, in addition to synonymous changes, base changes that resulted in between one and four amino acid substitutions (Fig. 1a; Table S2). Three of the clones contained single amino acid substitutions (Rd1-1 [K920E], Rd1-2 [Q931R], Rd1-3 [R618Q]) identifying these changes as being responsible for enhanced recognition. Interestingly, the single amino acid substitution K920E in R3a was recently also identified in a complementary study by Segretin *et al.* [25] as a substitution that enhances recognition towards AVR3a^EM^. The largest numbers of amino acid substitutions were found in LRRs #3 and #15 and included those found in the three clones with single substitutions (Fig. 1a).

A second round of shuffling was carried out to combine beneficial changes and remove deleterious substitutions using the eleven isolated clones, Rd1-1 to Rd1-11, and the wild-type gene as starting material. Screening of approximately six hundred recombinant clones identified four which gave responses comparable to or greater than the best performing clone from the first round, Rd1-1 [K920E]. All of these R3a* clones from the second round, Rd2-1 to Rd2-4, contained the amino acid substitution E620D in LRR #3 in addition to at least one other coding change (Fig. 1a; Table S2).

As recognition of AVR3a^EM^ improved and gave stronger responses it became more difficult to discriminate the differences in responses between modified clones with AVR3a^EM^ and also in comparison to the response of the wild-type *R3a* gene with AVR3a^KI^. Therefore, a third round of shuffling, using the clones from the first and second rounds of shuffling and the wild-type gene as starting material, was performed. However, the shuffled LRR domains were cloned in to a binary vector, pGRAB.R3a::R3a, containing the *R3a* gene or R3a* variants under the wild-type *R3a* promoter, rather than the strong 35S promoter. The purpose of this was to protract the timing of the cell death responses and facilitate the discrimination of differences (Fig. 1b). Screening of approximately 300 clones from this third round population identified three R3a* clones, Rd3-1 to Rd3-3, that gave responses greater than the best performing clone from the second round of shuffling, Rd2-1 [T585A, E620D]. All of these clones contained the E620D change found in the second round clones and a number of other amino acid changes including at least one change in LRR #15. A previously conducted comparison of functional R3a with three paralogous, non-functional resistance gene analogues revealed 13 positions under diversification [13]. These positions, with the exception of one in the CC-domain, are located in LRRs 1 to 4 and 14 to 23. Intriguingly, the majority of mutations present in the clones from the second and third rounds are within these LRRs (Fig. 1a).

One limitation of shuffling is in recombining beneficial mutations that are in close proximity due to the limited frequency of crossing-over. Therefore, as multiple mutations in LRRs #3 and #15 had been found to enhance AVR3a^EM^ recognition, an alternate approach was adopted. A population of site-directed mutants was produced using degenerate oligonucleotides that encoded different pairs of amino acids at positions 585 (T/A), 618 (R/Q), 620 (E/D), 918 (R/G), 920 (K/E), 923 (D/G) and 931 (Q/R) with the potential for 128 different permutations. Screening of approximately 400 clones from this population with the best performing clone from the third round of shuffling (Rd3-1 [E620D, L668P, Q931R]) as a reference, identified eight clones that produced responses with AVR3a^EM^ comparable to or greater than the reference. Five of these clones were found to have identical sequences. Representative unique clones, Rd4-1 to Rd4-4, contain the amino acid substitutions R618Q, E620D, K920E and Q931R, but lacked either of the designed substitutions R918G or D923G (Table S2).

### Iterative rounds of shuffling have progressively improved AVR3a^EM^ recognition by R3a* variants, producing faster and stronger PCD responses upon co-infiltration

The enhanced recognition of AVR3a^EM^ was assessed more accurately in single leaf comparisons. The R3a* constructs Rd2-1, Rd3-1 and Rd4-1 were transiently expressed under the control of the native *R3a* promoter with AVR3a^EM^ and the responses compared with those produced by the wild-type gene with AVR3a^EM^ and AVR3a^KI^. The best performing clone from the first round was not included in this analysis on account of the relatively poor response produced when it was under the control of the *R3a* promoter.

Symptom development on *N. benthamiana* was monitored for 9 days after infiltration in four independent experiments. In each experiment two adjacent, expanded leaves on each of twenty plants were infiltrated with the five infiltration mixtures in a circularly permuted arrangement to account for possible intra-leaf position effects. Symptoms were scored on an arbitrary scale ranging from 0 (no symptoms) to 10 (complete necrosis of the infiltrated area). A progressive increase in the recognition of AVR3a^EM^ was observed for the three R3a* clones with the necrotic response produced by Rd4-1 being close to that produced by the wild-type gene with AVR3a^KI^ (Fig. 1b).

A mixed model with experiment as a fixed effect was fitted to test for consistent responses of the infiltration mixtures over repeated experiments. The experiments showed significant differences in mean response (p = 0.001), but there was no significant interaction (p = 0.306) between experiment and infiltration mixture indicating that the relative responses of the phenotypes were stable over repeated experiments. In addition, there were no significant interactions between experiment and the other fixed effects.

Since the phenotypes were determined to be stable, a second analysis of the data with experiment as a random effect was now fitted. This showed highly significant effects (p<0.001) from infiltration mixture and leaf age (upper vs lower leaf). Further, there was a highly significant interaction (p<0.001) between infiltration mixture and leaf age because the combination of R3a and AVR3a^EM^ did not show a difference in mean scores between younger and older leaves, in contrast to the other combinations which showed significant differences (Fig. 1c). Position of infiltration site on leaf was non-significant and all other interactions of fixed effect were also non-significant. Multiple comparisons using Bonferroni correction with an experiment-wise significance level of 5% showed significant differences between the mean symptom scores for all infiltration mixtures within either younger or older leaves with a comparison-wise significance level of 0.0011 (Table S3). Ordering of the responses was the same for both younger and older leaves with R3a and AVR3a^KI^> Rd4-1 & AVR3a^EM^> Rd3-1 & AVR3a^EM^> Rd2-1 & AVR3a^EM^> R3a and AVR3a^EM^. While the clone Rd2-1 from the second round gave symptoms when co-expressed with AVR3a^EM^, it rarely produced an HR phenotype as shown in figure 1d, which shows the proportion of sites with more than 50% of the infiltrated area necrotic. Despite improved recognition of AVR3a^EM^ by the R3a* variants relative to wild-type R3a, their recognition of AVR3a^KI^ was not impaired (data not shown), indicating that this specificity had not been significantly attenuated. Further, when expressed from the strong 35S promoter in the absence of AVR3a^EM^ or AVR3a^KI^ none of the clones produced necrosis, indicating that they maintained appropriate control mechanisms and were not auto-activators (Fig. S1).

### R3a* recognition of Avr3a^EM^ is dependent on HSP90 and SGT1

**Figure 2:**
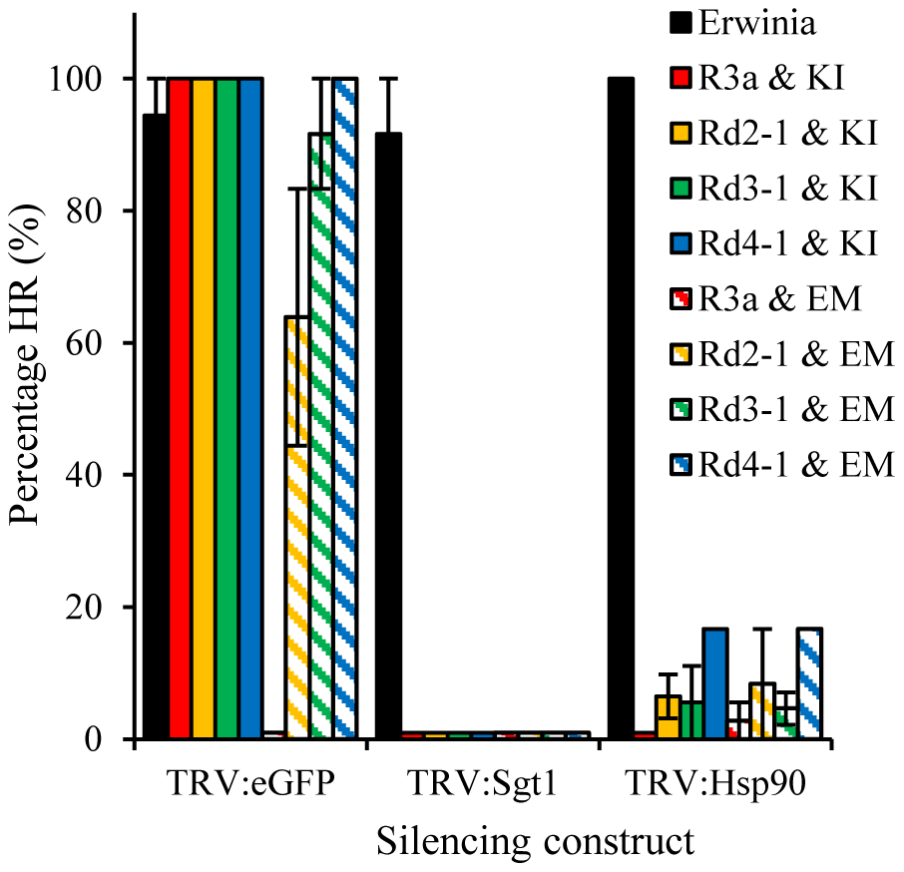
HR responses resulting from R3a* recognition of AVR3a^EM^ and AVR3a^KI^, like those caused by wild-type R3a recognition of AVR3a^KI^, are dependent on SGT1 and HSP90. SGT1- and HSP90-silenced plants were produced using TRV-based vectors. These plants and control plants inoculated with TRV:*eGFP* were infiltrated with different combinations of *Agrobacterium* cultures designed to express R3a, R3a* variants, AVR3a^KI^ (KI) or AVR3a^EM^ (EM). The percentage of sites (N = 12) showing HR responses six days after infiltration was recorded. The graph shows the mean percentages from three independent experiments with the exception that the dependence on HSP90 of Rd4-1 responses was only tested in a single experiment. The non-host bacterial pathogen *Erwinia amylovora* was used as a control for an SGT1- and HSP90-independnet HR response. Error bars show +/− standard error. Zero values have been transformed to 1% to facilitate their observation.

Previous studies by Bos *et al.* [14] demonstrated that R3a-dependent recognition of AVR3a^KI^ involves both SGT1 (suppressor of the G2 allele of *skp1*) and HSP90 (heat shock protein 90) that are required for the activation of other R proteins [26]–[27]. Their involvement in the AVR3a^EM^-dependent responses was tested through *Tobacco rattle virus* (TRV)-based gene silencing of *SGT1*and *HSP90* in *N. benthamiana* with TRV-based expression of truncated GFP (eGFP) as a control. Three biological replicates for R3a, Rd2-1, Rd3-1 and one for Rd4-1, with infiltrations in two leaves of each of six plants per TRV-based silencing construct, revealed that both SGT1 and HSP90 are required to mediate an HR upon R3a*-based recognition of AVR3a^KI^ and AVR3a^EM^ (Fig. 2, Fig. S2). HRs were abolished for all infiltrations on TRV:*SGT1* inoculated plants and there were almost no HRs recorded on TRV:*HSP90* inoculated plants (Fig. 2). The HRs were not affected on plants inoculated with TRV:*eGFP*.

Compared to TRV:*eGFP* inoculated plants, *SGT1* and *HSP90* silenced plants were morphologically stunted, a phenotype that had been reported previously [14]. Nevertheless, upon infection with the bacterial pathogen *Erwinia amylovora* that produces a SGT1 and HSP90 independent non-host response in *N. benthamiana* [28], all plants were able to mount the expected HR response (Fig. 2, Fig. S2).

### In a gain of mechanism, R3a* variants re-localize to late endosomes upon co-infiltration with Avr3a^KI^ or Avr3a^EM^

In a previous study, Engelhardt *et al.* [15] demonstrated that, upon recognition of AVR3a^KI^ but not AVR3a^EM^, wild-type R3a re-localizes from the host cytoplasm to specific late endosomes that can be labelled with the cyan fluorescent protein marker PS1-CFP [29]. This re-localization was found to be a pre-requisite for subsequent HR development for untagged R3a co-expressed with AVR3a^KI^ [15]. To study if R3a* variants with enhanced recognition of AVR3a^EM^ had gained the capacity to re-localize upon detection of AVR3a^EM^ and continued to exhibit this phenotype following detection of AVR3a^KI^, N-terminal fusions of R3a* variants Rd2-1, Rd3-1 and Rd4-1 with yellow fluorescent protein (YFP) were generated as described previously with expression of these constructs driven by a 35S promoter. Western-blot analysis of protein extracts from inoculated tissue demonstrated the integrity of the fusion proteins (Fig. S3). As demonstrated for YFP-R3a wild-type fusions by Engelhardt *et al.* [15], YFP-R3a* fusions did not elicit HRs alone or in the presence of AVR3a^KI^ or AVR3a^EM^, probably due to steric hindrance of the signalling domains of R3a (data not shown).

**Figure 3:**
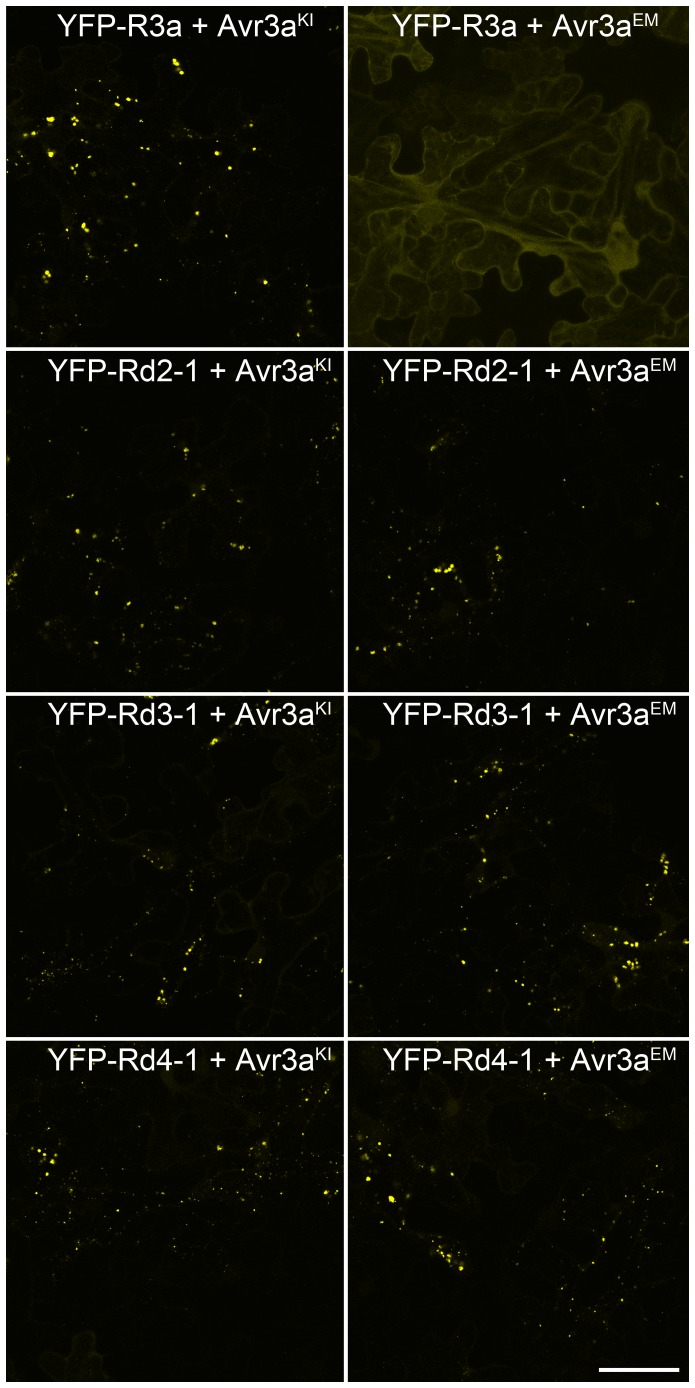
YFP fusions to R3a* variants re-localize to vesicles after the perception of both of AVR3a^KI^ and AVR3a^EM^, whereas YFP-R3a remains cytoplasmic in the presence of AVR3a^EM^. Two days after infiltration of mixtures of *Agrobacterium* cultures designed to express AVR3a^KI^, AVR3a^EM^, YFP-R3a or YFP fusions to the R3a* variants, infiltrated *N. benthamiana* leaf tissue was examined under a confocal laser scanning microscope. Scale bar  = 50 µm.

As anticipated, following transient expression in *N. benthamiana*, all YFP-R3a/R3a* fusions when expressed by themselves displayed cytoplasmic localizations (Fig. S4). In accord with the observations described by Engelhardt *et al.* [15], the localisation of YFP-R3a remained cytoplasmic upon co-infiltration with AVR3a^EM^ (Fig. 3), but changed to fast moving, PS1-CFP labelled vesicles, following recognition of AVR3a^KI^. The YFP-R3a* fusions of Rd2-1, Rd3-1 and Rd4-1 proteins maintained this mechanistically characteristic re-localisation following co-expression with AVR3a^KI^ (Fig. 3). However, in contrast to YFP-R3a, all selected YFP-R3a* variants also displayed highly reproducible re-localization to PS1-CFP labelled vesicles after the perception of AVR3a^EM^ (Fig. 3; Fig S5).

### AVR3a^KI^ and AVR3a^EM^ re-localize to endosomes upon co-infiltration with R3a* variants but not, in the case of AVR3a^EM^, with wild-type R3a

As shown by Engelhardt *et al.* [15] AVR3a^KI^, but not AVR3a^EM^, also re-localizes from the cytoplasm to endosomes upon co-expression with R3a. This was demonstrated by N-terminal fusions of AVR3a^KI^ and AVR3a^EM^ to green fluorescent protein as well as by BiFC, also known as split-YFP, assays [9], [15], [30]. The latter revealed that wild-type R3a and AVR3a^KI^ are found in close proximity at PS1-CFP labelled vesicles [15].

To investigate if the vesicular co-association of R3a and AVR3a^KI^ was extended to the R3a* variants, BiFC was used to analyse and localize protein–protein interactions *in planta*. As described previously for wild-type R3a, the N-terminal portion of YFP, YN, was fused to the N-terminal end of the R3a* variants Rd2-1, Rd3-1 and Rd4-1. The constructs used to express the C-terminal portion of YFP, YC, fused to AVR3a^KI^ and AVR3a^EM^ were as described previously [15] with all constructs being transiently expressed in *N. benthamiana* from the 35S promoter.

**Figure 4:**
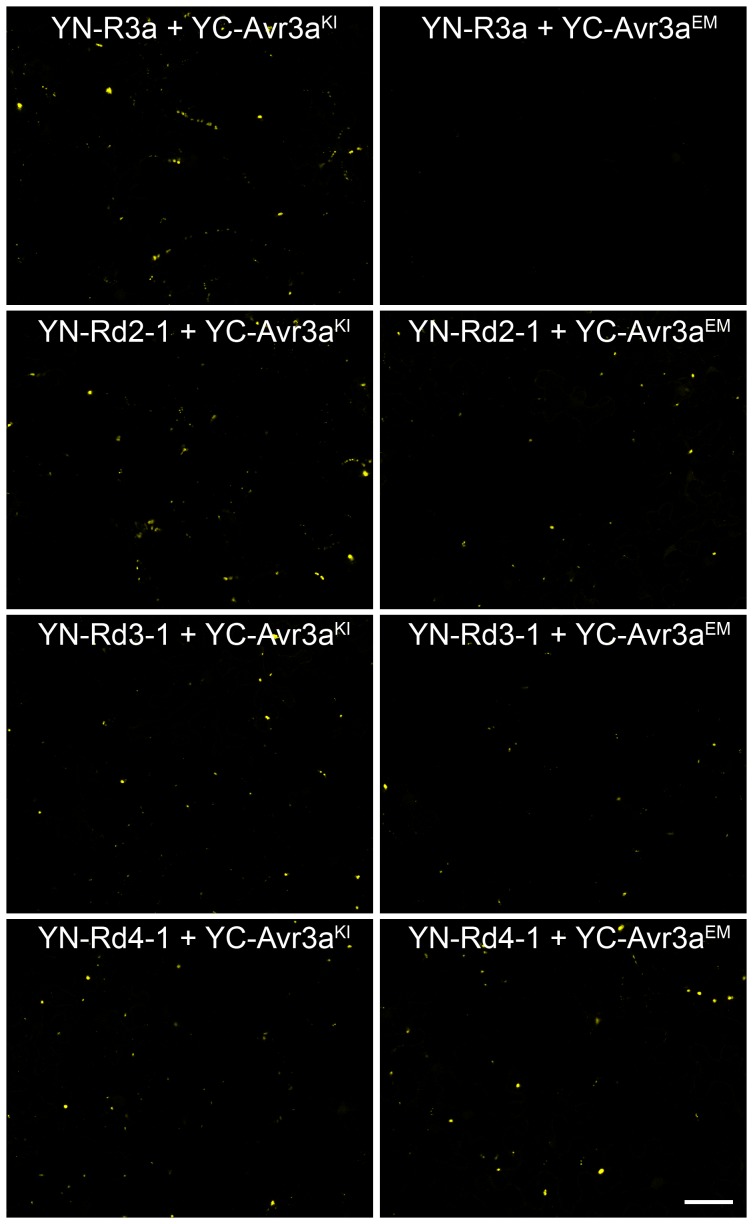
Both YC-AVR3a^KI^ and YC-AVR3a^EM^ when co-expressed with YN-R3a* fusions give vesicle associated YFP fluorescence like YC-AVR3a^KI^ and YN-R3a, whereas YC-AVR3a^EM^ and YN-R3a do not. Two days after infiltration of mixtures of *Agrobacterium* cultures designed to express YC-AVR3a^KI^, YC-AVR3^EM^, YN-R3a or YN fusions to the R3a* variants, infiltrated *N. benthamiana* leaf tissue was examined under a confocal laser scanning microscope. Representative images from two experiments. Scale bar  = 50 µm.

In accord with previous findings [15], co-expression of YN-R3a with YC-AVR3a^KI^ gave strong YFP fluorescence, whereas co-expression with YC-AVR3a^EM^ did not give detectable YFP fluorescence (Fig. 4). Like the YN fusion to wild-type R3a, all the YN-R3a* fusions when co-expressed with AVR3a^KI^ gave strong, punctate, YFP signals (Fig. 4), but unlike the wild-type fusion also gave YFP fluorescence signals at PS1-CFP labelled vesicles when co-expressed with AVR3a^EM^ (Fig. 4; Fig. S6). This indicates that AVR3a^EM^ is also within close proximity of the re-localized R3a* gene products. Thus, in line with the gain of recognition of AVR3a^EM^ by the R3a* variants and subsequent necrosis responses, the R3a* variants and AVR3a^EM^ show the same mechanistic re-localization as observed for R3a and AVR3a^KI^.

### R3a* variants maintain resistance towards AVR3a^KI^-expressing *P. infestans* isolates but have not gained resistance towards AVR3a^EM^ homozygous isolates

To evaluate if R3a* variants with gain of AVR3a^EM^ recognition and re-localisation mechanism yield effective disease resistance, transient and stable expression systems were utilised. *Agrobacterium tumefaciens* transient assays (ATTAs) in *N. benthamiana* have successfully been used to demonstrate function for late blight resistance gene products such as R2, Rpi_STO1 [31] and R3b [32]. Selected R3a* clones Rd2-1, Rd3-1 and Rd4-1 were transiently expressed in *N. benthamiana* using the *R3a* promoter in ATTAs alongside wild-type R3a and an empty vector control. Infiltrated leaf areas were challenged two days after infiltration with AVR3a^KI^ or AVR3a^EM^ homozygous *P. infestans* isolates via drop inoculation. Disease progression was monitored by measuring visible lesion diameters in multiple independent experiments and analysis of variance was carried out using GenStat on the data from individual experiments to test for significant differences. Multiple comparisons were performed using Bonferroni correction with a significance level of 5% and a comparison-wise error rate of 0.005.

**Figure 5:**
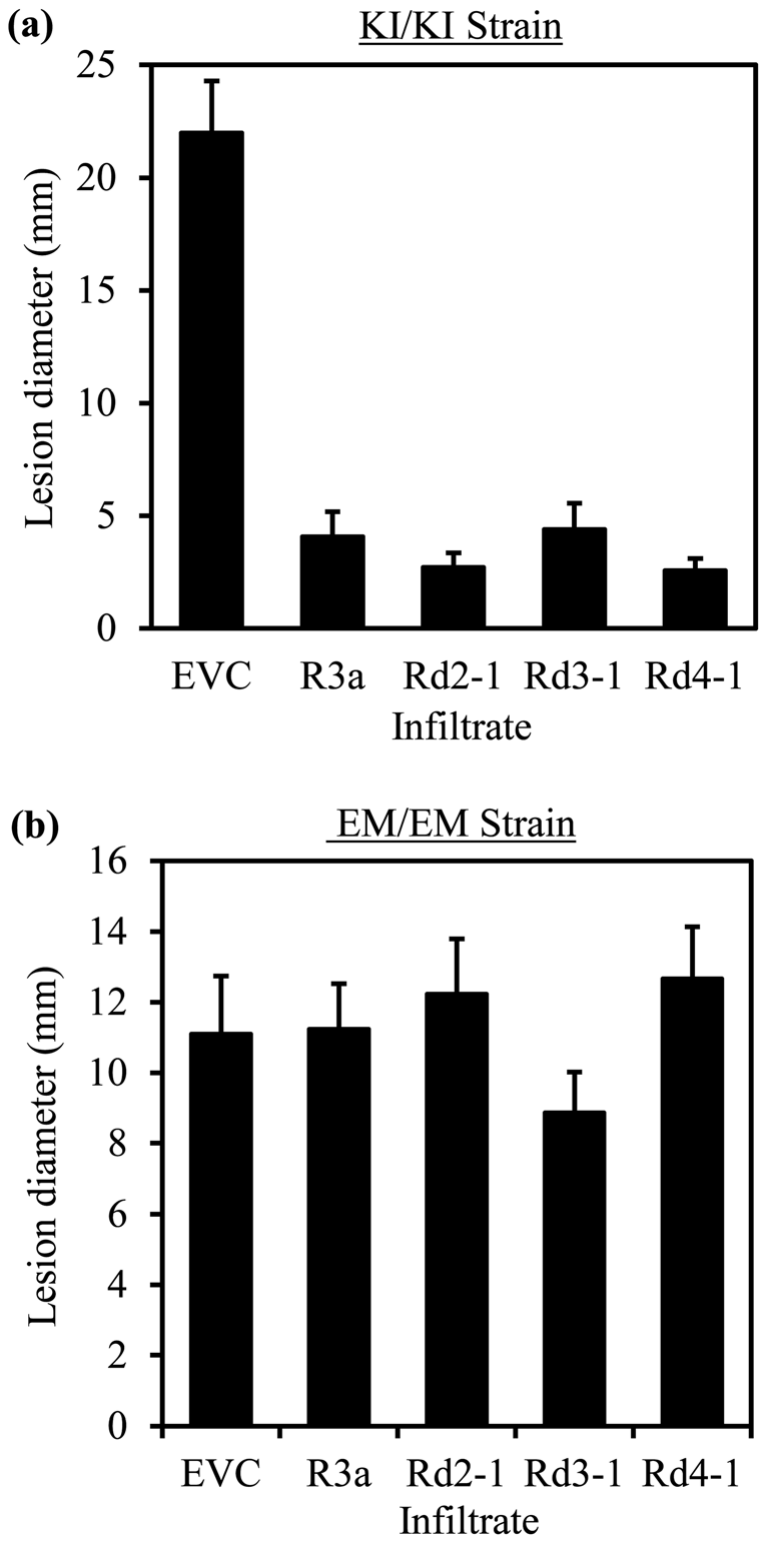
R3a and R3a* variants expressed from *Agrobacterium* reduce the spread of a *P. infestans* strain expressing AVR3a^KI^, but not the spread of a strain expressing only AVR3a^EM^. (a) Means of lesion diameters measured 12 days after drop inoculation of agro-infiltrated areas with strain 7804.b (KI/KI). (b) Means of lesion diameters measured 8 days after drop inoculation of agro-infiltrated areas with strain 88069 (EM/EM). (a) and (b) show representative experiments from sets of three and five repeated experiments, respectively. For both (a) and (b), error bars show +/− standard errors, N = 30. EVC indicates empty vector control.

In three experiments ATTA sites were inoculated with the AVR3a^KI^ homozygous *P. infestans* isolate 7804.b. In all three experiments the wild-type R3a and the R3a* variants significantly reduced spread of *P. infestans* relative to the empty vector control and there were no significant differences between the different R3a forms (Fig. 5a). This result indicates that the selected mutations in the LRR do not impair the resistance induced by AVR3a^KI^. Likewise ATTA sites were inoculated with the AVR3a^EM^ homozygous isolate 88069 [9] in five experiments. In four of the five experiments there were no significant differences in *P.* infestans spread between any of the R3a forms and the empty vector control (Fig. 5b). In the fifth experiment there was significantly increased spread with the empty vector control, but the R3a* variants showed no significant differences from the wild-type gene which does not provide resistance against AVR3a^EM^ homozygous isolates. Co-infiltrations of R3a, R3a*, AVR3a^KI^ and AVR3a^EM^ constructs were carried out contemporaneously in all experiments to confirm that the conditions were conducive to HR development (data not shown).

**Figure 6:**
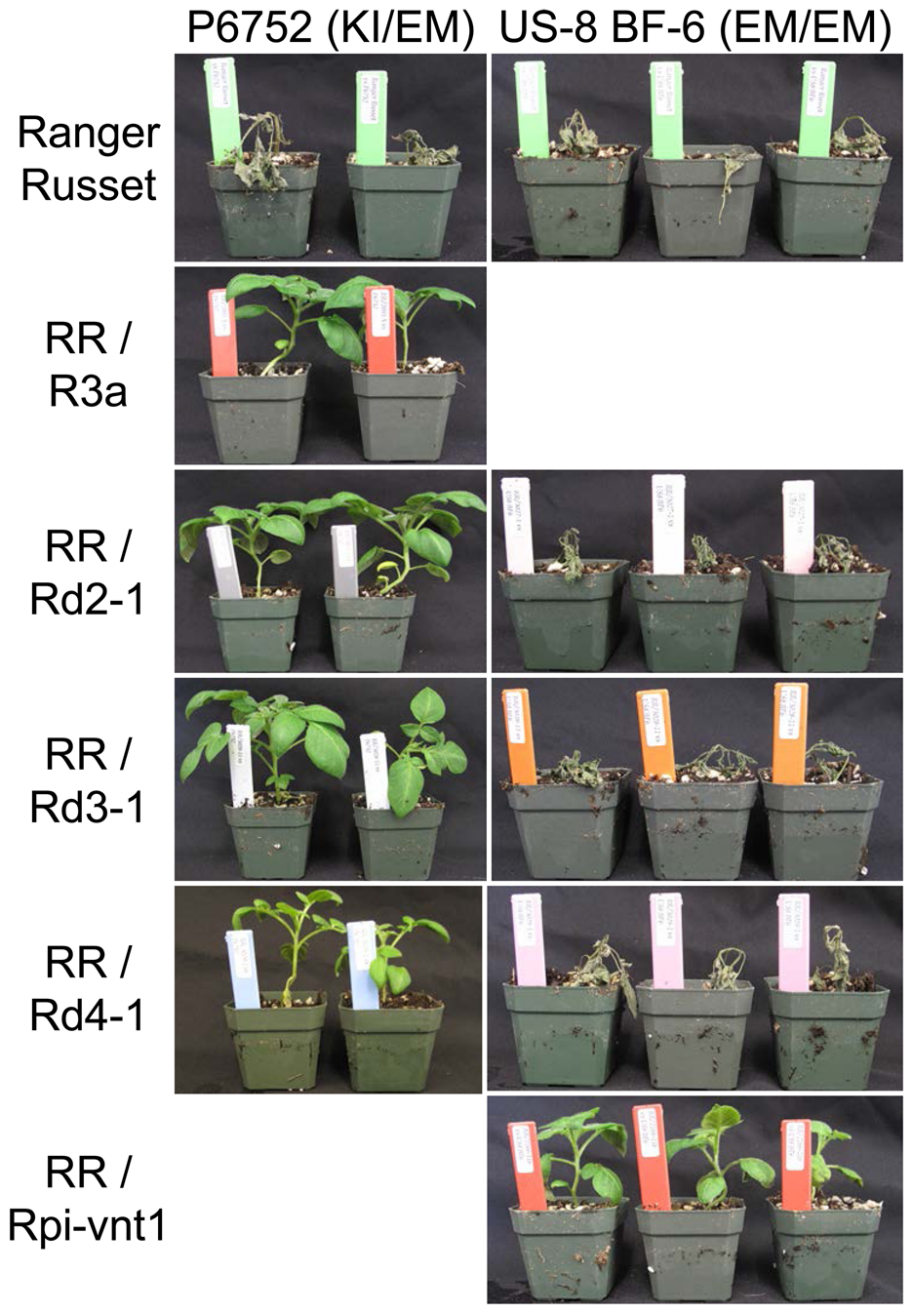
R3a and R3a* variants expressed via the *R3a* promoter in transgenic plants protect the susceptible cultivar Ranger Russet from *P. infestans* strain P6752, which is heterozygous for AVR3a^KI^ and AVR3a^EM^, but not from strain US-8 BF-6, which is homozygous for AVR3a^EM^. Non-transgenic plants were used as a control for susceptibility. Transgenic plants expressing R3a or Rpi-vnt1 were used as positive controls for resistance to P6752 or US-8 BF-6, respectively. Representative plants were photographed at 11dpi.

To confirm these results and to rule out potential adverse effects and limitations of the ATTA system in *N. benthamiana*, transgenic potato plants were generated. The wild-type *R3a* gene and the R3a* genes for Rd2-1, Rd3-1 and Rd4-1 were transformed into the potato cultivar Ranger Russet using the *R3a* promoter and terminator to regulate gene expression and stability. Transgenic Ranger Russet lines expressing R3a and the three R3a* variants were compared to Ranger Russet lines containing the *Rpi_vnt1* gene [33] and non-transgenic Ranger Russet plants as positive and negative controls, respectively. Transgenic lines were challenged with the Mexican isolate P6752, which is heterozygous for AVR3a^KI^ and AVR3a^EM^, and the US isolate US-8 BF-6, which is homozygous for AVR3a^EM^. The transgenic potato plants expressing R3a, Rpi_vnt1 and the R3a* variants, but not the non-transgenic Ranger Russet, demonstrated high levels of resistance towards the heterozygous isolate P6752 (Fig. 6). Thus, the transient ATTA data and the transgenic plants corroborate the conclusion that the selected mutations in the LRR do not negatively impact on resistance towards *P. infestans* isolates expressing Avr3a^KI^. The transgenic Ranger Russet lines expressing Rpi_vnt1 provided resistance towards the AVR3a^EM^ homozygous isolate US-8 BF-6. However, none of the transgenic lines expressing the R3a* or *R3a* genes, which had been shown to be resistant to isolate P6752, were able to control disease development of isolate US-8 BF-6 (Fig. 6).

## Discussion

The relatively narrow genetic basis of clonal potato cultivars in agriculture provides pathogens such as *P. infestans* with sufficient opportunity to adapt and overcome inducible host resistant responses and to thus cause disease on a global scale. Resistance responses rely on the direct or indirect recognition of modified-self or pathogen-derived molecules [1]. For example, in the first layer of inducible resistance, also referred to as PAMP triggered immunity (PTI), conserved pathogen-associated molecular patterns (PAMPs) and/or damage-associated molecular patterns (DAMPs) are recognised [1], [34], [35]. Successful pathogens circumvent this recognition with the help of effectors that perturb host resistance responses and promote effector-triggered susceptibility (ETS). However, by being in close proximity to the host, pathogen effectors provide the innate plant immune system with another opportunity for detection that is dependent on the presence of cognate plant R proteins that subsequently yield ETI [1], [36]. In nature, this closely entwined co-evolution between hosts and pathogens is evident in the diversification observed for effectors and *R* genes [4].

Indeed, *P. infestans* is known as a pathogen with ‘high evolutionary potential’ [37] and more than 560 RXLR-type candidate effectors have been described within the late blight pathogen genome [38]. The genomic organisation of RXLR effector genes, which are often in gene-poor and repeat-rich regions, is thought to facilitate their enhanced diversification by enabling non-homologous recombination. Oomycete RXLR-type effectors have been shown to evade detection by *R* gene products via transcriptional regulation [39], utilising functionally equivalent effectors that allow loss of recognised effectors [40], suppressor activity of unrelated effectors [41] and/or sequence diversity [11], [42]. Sequence diversity underpins virulence or avirulence behaviour for the essential *P. infestans* effector AVR3a. AVR3a^KI^ determines avirulence on plants carrying *R3a* whereas AVR3a^EM^ promotes virulence. It is thought that only AVR3a^KI^ was present in the *P. infestans* strain responsible for the outbreak of late blight disease leading to the Irish Potato Famine in the 1840 s [43]. The AVR3a^EM^ allele may have come to dominate in *P. infestans* populations once the resistance gene *R3a* was deployed in potato crops, quickly usurping the AVR3a^KI^ allele. By using clonal potato varieties in current agriculture, the diversification of *R* genes is undermined and novel, naturally occurring resistances can only slowly be introgressed via breeding.

Functional *R* genes are often found in clusters that show evidence of duplication followed by diversification [44]. The phylogenetic NB-LRR gene grouping that contains homologs of the functional *R3a* gene in the potato genome has previously been described as CNL-8 [5]. This group is, after the *R2* cluster (CNL-5), the second largest *R* gene cluster in potato where more than 750 NB-LRR-like genes have been identified [5]–[6]. Functional *R3a*, a homolog of the tomato *I2* resistance gene that controls races of the fungus *Fusarium oxysporum* [45], was cloned alongside three paralogous sequences that provided insight into amino acid positions under diversification [13]. With one exception, positions under diversification reside in the LRR and cluster around two regions spanning LRRs 1 to 4 and 14 to 23 that were also identified as being important in this study.

Here we used, in addition to random mutagenesis and screening, DNA shuffling and targeted mutagenesis to enhance AVR3a^EM^ recognition by R3a*. DNA shuffling, which emulates the natural evolutionary processes of mutation, recombination and selection, has proven a highly effective method for evolving new specificities/properties for a wide range of proteins that cannot be rationally designed and is of particular use in identifying mutations that are beneficial in combination [18]. Artificial evolution of a resistance gene to broaden its specificity has previously been performed on the gene *Rx* that protects potato plants from strains of PVX. In an initial *Rx* study, mutagenesis of the LRR region was performed on the basis that this region is the primary determinant of recognition specificity. Screening identified four mutations in the LRR region that affected elicitor recognition and activation functions [16]. Introduction of the mutated *Rx* genes in to the model host *N. benthamiana* as transgenes extended resistance to a normally resistance-breaking strain of PVX, HB, and a distantly related carlavirus, PopMV [16].

Our primary screen identified eleven mutants with enhanced AVR3a^EM^ recognition containing in total 23 amino acid substitutions. However, only three of these (R618Q, K920E, Q931R), found in the clones with single amino acid substitutions, are known to be causative to the improved phenotype. The previous study performed by Segretin *et al.* [25] identified six amino acid substitutions in the LRR domain that enhanced AVR3a^EM^ recognition: two of these were also found in our primary screen (L668P, K920E). The fact that we did not identify all the LRR mutations identified by Segretin *et al.* [25] and that they did not identify more mutations in the LRR region, demonstrates that neither screen was exhaustive.

The initial screen identified mutants with enhanced recognition of AVR3a^EM^ when expressed from the strong 35S promoter. Amino acid changes in LRRs #3 and #15, and thus within regions known to be under diversifying selection [13], were prevalent in the clones from the first round of screening, suggesting these regions might be of particular importance. This suggestion was supported by the fact that all the clones from the second round of shuffling contained an amino acid change, E620D, in LRR #3 and sometimes additional changes in LRR #15, while all of the clones from the third round contained the E620D change and one or two amino acid changes in LRR #15. Using DNA shuffling it is more difficult to bring together combinatorially beneficial mutations that are in close proximity. Furthermore, random mutagenesis as a source of diversity has limitations in that single nucleotide changes can only convert a codon for one amino acid to a limited set of codons for other amino acids rather than all twenty possible amino acids. To circumvent the former problem a more directed approach was adopted in which a library that contained all permutations of the amino acid changes thought important (one in LRR #2, two in LRR #3 and four in LRR #15) was produced for screening. The aptness of this approach was shown by the fact that clones were obtained with enhanced recognition responses to AVR3a^EM^ compared to the best performing clone from the third round of shuffling, Rd3-1, and that one of the possible 128 forms was prevalent. This form contained two amino acid changes in close proximity in each of LRRs #3 and #15; a combination that would have been difficult to obtain through DNA shuffling. Interestingly, an amino acid change at one of these positions, Q931, was also found in one of the clones recovered by Segretin *et al.* [25], though their “de-convolution” did not show their substitution at this position, proline instead of arginine, to improve AVR3a^EM^ recognition. As shown by Segretin *et al.* [25] for the R3a mutants with enhanced AVR3a^EM^ recognition, we did not note any reduction in the AVR3a^KI^ recognition responses of the clones we isolated.

Our studies show that the recognition of AVR3a^EM^ by the R3a* mutants we have isolated recapitulates the mechanistic processes of recognition of AVR3a^KI^ by the wild-type *R3a* gene. It has previously been reported [14] that the HR triggered by R3a recognition of AVR3a^KI^ is dependent on the ubiquitin ligase-associated protein SGT1 and HSP90. VIGS of *SGT1* and, to a lesser degree, of *HSP90* in our experiments inhibited the cell death responses induced by recognition of AVR3a^EM^ by our R3a* mutants. Similarly, it has been shown that wild-type R3a re-localises from the cytoplasm to late endosomal compartments when co-expressed with AVR3a^KI^, but not when co-expressed with AVR3a^EM^ [15]. We found that the mutants from the three later rounds still re-localised to endosomal compartments when co-expressed with AVR3a^KI^ and, importantly, also re-localised to the same vesicles when co-expressed with AVR3a^EM^. The earlier study by Engelhardt *et al.* [15] also showed that the effector AVR3a^KI^ itself relocalises from the cytoplasm to endosomes when co-expressed with wild-type R3a and is in close physical proximity to R3a, whereas AVR3a^EM^ remains distributed through the cytoplasm. Our BiFC experiments show that AVR3a^KI^ and the normally unrecognized form, AVR3a^EM^ both traffic from the cytoplasm to vesicles when co-expressed with the R3a* forms. This re-localisation of R3a and AVR3^KI^ was shown to be a prerequisite for the development of the HR [15]. Thus, the gain of AVR3a^EM^ recognition R3a* variants have gained many aspects of the mechanism of the wild-type R3a response to AVR3a^KI^.

Although the R3a* variants produced in this study responded to AVR3a^EM^ and produced HR responses when the elicitor was transiently expressed via *Agrobacterium*, critically they only provided resistance to *P. infestans* isolates that express AVR3a^KI^ and not to isolates that express only AVR3a^EM^. Both transient expression via *Agrobacterium* in *N. benthamiana* and stable transgenic expression in potato corroborated this finding. Failure to protect from the pathogen itself was also reported for the R3a mutants identified by Segretin *et al.* [25]. That this was the case for mutants with single amino acid changes is perhaps not surprising given the large differences from the wild-type R3a/AVR3a^KI^ response we observed when first and second round clones were expressed from the *R3a* promoter with AVR3a^EM^. For some pathogen/*R* gene combinations, e.g. PVX and *Rx*, the resistance responses can be separated from the HR [46] though the induction of necrotic responses by transient expression of the elicitor protein has been used to identify Rx mutations that, when expressed transgenically in the model host *N. benthamiana*, provide resistance to the pathogen itself. However, for *P. infestans* it has been suggested that the strength of the HR correlates with resistance levels [47].

A recent, secondary mutation study of *Rx* provides some evidence that stepwise artificial evolution could be required to obtain an optimum combination between effector recognition and subsequent *R* gene activation and signal transduction [17]. In the *Rx* study, the resistance provided by one of the mutations in the LRR domain to PopMV was improved by random mutagenesis of the CC-NB-ARC1-ARC2 domains [17]. In addition to constitutively active mutants that by themselves gave necrotic responses, four mutants with enhanced responses to PopMV were isolated. For three of these mutants the improved phenotype was conferred by a single amino acid change, while for the fourth a pair of amino acid substitutions was required. The mutations, which affect activation sensitivity, were found to be located around the nucleotide-binding pocket of Rx. As mentioned previously, we have evidence that neither this screening nor the efforts from Segretin *et al.* [25] were exhaustive as both approaches yielded novel beneficial mutations. Whereas our study of *R3a* focused solely on the LRRs, in Segretin *et al.* [25] the entire *R3a* gene was subjected to mutagenesis. The latter study identified eight single amino acid changes that enhanced responses to AVR3a^EM^. Out of these, six occurred in the LRR domain and one in the CC domain. This substitution enhanced the response to AVR3a^EM^ but also showed some auto-activation. The final substitution, found in the NB-ARC domain, sensitised the AVR3a^EM^ response and broadened specificity to include an elicitor from another *Phytophthora* species [25]. Interestingly, this change occurred in the nucleotide-binding pocket and is adjacent to one of the sensitizing mutations found in *Rx* [17]. Broadening resistance gene specificity merely by introducing sensitizing mutations without improving recognition may have detrimental consequences in the field. However, a natural precedent for this has been found in PM3 resistance protein alleles in which the substitution of two amino acids in the NB domain enhances the HR and broadens the spectrum of resistance to wheat powdery mildew isolates [48]. Thus, additional efforts to combine novel mutations in R3a domains responsible for AVR3a^EM^ recognition (LRR) and response (CC-NB-ARC1/ARC2) could further improve R3a* variants that already display gain of recognition and mechanistic re-localisation to ultimately yield genes that provide effective resistance in potato against isolates expressing AVR3a^EM^. Considering the importance of AVR3a to *P. infestans*, such a resistance, combined with other, mechanistically distinct *R* genes could provide a step towards more durable late blight control.

## Supporting Information

Figure S1 R3a* variants are not auto-activators. *N. benthamiana* leaves were infiltrated with *Agrobacterium* cultures designed to express R3a or the R3a* variants from the strong 35S promoter. Mixtures of cultures designed to co-express AVR3a^KI^ (KI) were used as positive controls for the induction of cell death. Leaves were examined under white-light and UV-B illumination. Photograph of representative leaf was taken five days after infiltration. In the absence of elicitor the R3a* variants, like R3a, do not produce visible cell death.
(TIF)

Figure S2 HR responses resulting from R3a* recognition of AVR3a^EM^ and AVR3a^KI^, like those caused by wild-type R3a recognition of AVR3a^KI^, are dependent on SGT1 and HSP90. SGT1- and HSP90-silenced plants were produced using TRV-based vectors. These plants and control plants inoculated with TRV:*eGFP* were infiltrated with different combinations of *Agrobacterium* cultures designed to express R3a, R3a* variants, AVR3a^KI^ (KI) or AVR3a^EM^ (EM). Photographs show representative HR responses induced by each of the different mixtures on control TRV:eGFP inoculated plants, SGT1-silenced plants and HSP90-silenced plants. The non-host bacterial pathogen *Erwinia amylovora* was used as a control for an SGT1- and HSP90-independnet HR response.
(TIF)

Figure S3 **Western blot analysis showing integrity of YFP fusion proteins.** Soluble protein extracts were prepared from *N. benthamiana* leaf tissue two days after infiltration with cultures designed to express YFP fusions to R3a, Rd2-1, Rd3-1 or Rd4-1. The blot was probed with anti-GFP antibodies as described by Engelhardt *et al*. (2012). Protein sizes are indicated in kilodaltons (kD) and protein loading is shown by Ponceau S (PS) staining.
(TIF)

Figure S4 YFP fusions to R3a and the R3a* variants localize to the cytoplasm in the absence of AVR3a. *N. benthamiana* leaves were infiltrated with cultures designed to express YFP fusions to R3a, Rd2-1, Rd3-1 or Rd4-1. Leaf tissue was examined two days after infiltration under a confocal laser scanning microscope. Representative images are from five independent experiments. Scale bar  = 20 µm.
(TIF)

Figure S5 In the presence of AVR3a^EM^ YFP fusions to R3a* variants, but not YFP-R3a, re-localize to vesicles labelled by the prevacuolar compartment marker PS1-CFP. *N. benthamiana* leaves were infiltrated with mixtures of cultures designed to express PS1-CFP, AVR3a^EM^ and YFP fusions to R3a, Rd2-1, Rd3-1 or Rd4-1. Tissue was examined two days after infiltration under a confocal laser scanning microscope. The left-hand panel shows YFP signal, the right-hand panel CFP signal and the central panel displays the merged signals. Representative images are from three independent experiments. Scale bar  = 10 µm.
(TIF)

Figure S6 YC-AVR3a^EM^ reconstitutes YFP fluorescence with YN fusions to the R3a* variants at vesicles labelled by the prevacuolar compartment marker PS1-CFP. Generation of the YFP signal indicates that AVR3aEM and the R3a* variants are in close proximity at the vesicles. *N. benthamiana* leaves were infiltrated with mixtures of cultures designed to express PS1-CFP, YC-AVR3a^EM^ and YN fusions to Rd2-1, Rd3-1 or Rd4-1. Tissue was examined 2 d after infiltration under a confocal laser scanning microscope. Left-hand panel, YFP signal; right-hand panel, CFP signal; central panel, merged signals. Representative images from three experiments. Scale bar  = 20 µm.
(TIF)

Table S1 **Primers used in this study.**
(DOCX)

Table S2 Nucleotide and amino acid changes found in R3a* clones.
(DOCX)

Table S3 **Mean symptom scores from four nine-day experiments for upper and lower leaves.**
(DOCX)
