# Supplementary figures and images for: Detection of the Virulent Form of AVR3a from Phytophthora infestans following Artificial Evolution of Potato Resistance Gene R3a
Source: PLoS One. 2014 Oct 23;9(10):e110158. doi: 10.1371/journal.pone.0110158 (PMC4207746; doi:10.1371/journal.pone.0110158)

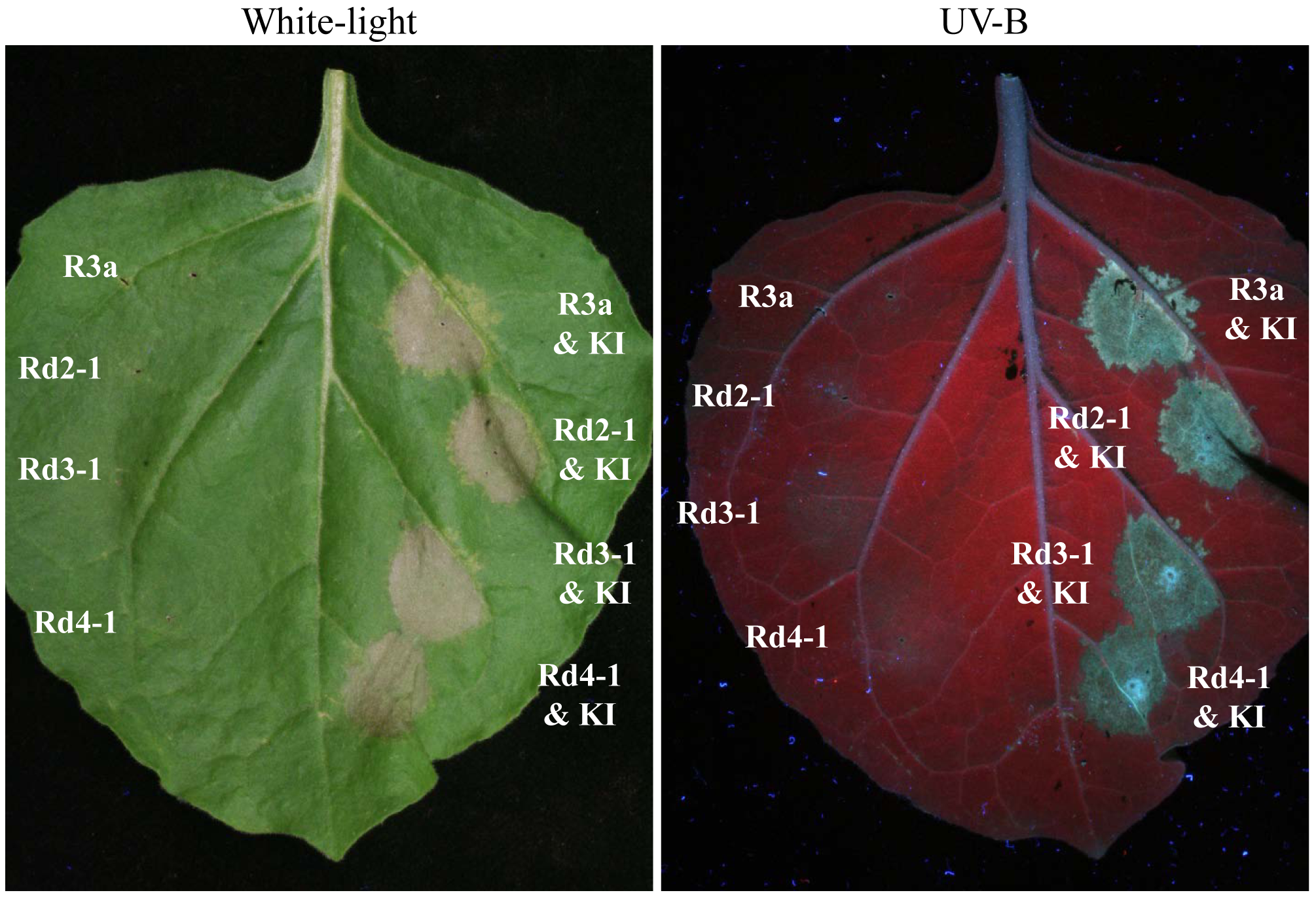

Supplement: Figure S1 — R3a* variants are not auto-activators. N. benthamiana leaves were infiltrated with Agrobacterium cultures designed to express R3a or the R3a* variants from the strong 35S promoter. Mixtures of cultures designed to co-express AVR3aKI (KI) were used as positive controls for the induction of cell death. Leaves were examined under white-light and UV-B illumination. Photograph of representative leaf was taken five days after infiltration. In the absence of elicitor the R3a* variants, like R3a, do not produce visible cell death. (TIF) [file pone.0110158.s001.tif]

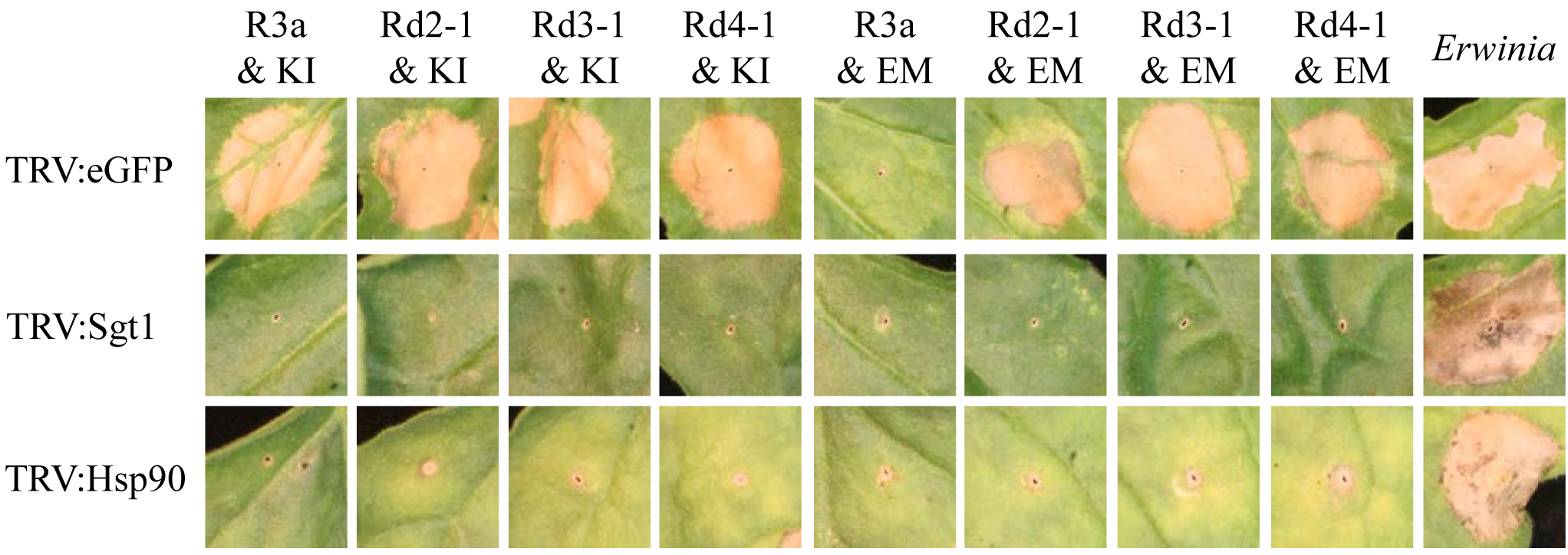

Supplement: Figure S2 — HR responses resulting from R3a* recognition of AVR3aEM and AVR3aKI, like those caused by wild-type R3a recognition of AVR3aKI, are dependent on SGT1 and HSP90. SGT1- and HSP90-silenced plants were produced using TRV-based vectors. These plants and control plants inoculated with TRV:eGFP were infiltrated with different combinations of Agrobacterium cultures designed to express R3a, R3a* variants, AVR3aKI (KI) or AVR3aEM (EM). Photographs show representative HR responses induced by each of the different mixtures on control TRV:eGFP inoculated plants, SGT1-silenced plants and HSP90-silenced plants. The non-host bacterial pathogen Erwinia amylovora was used as a control for an SGT1- and HSP90-independnet HR response. (TIF) [file pone.0110158.s002.tif]

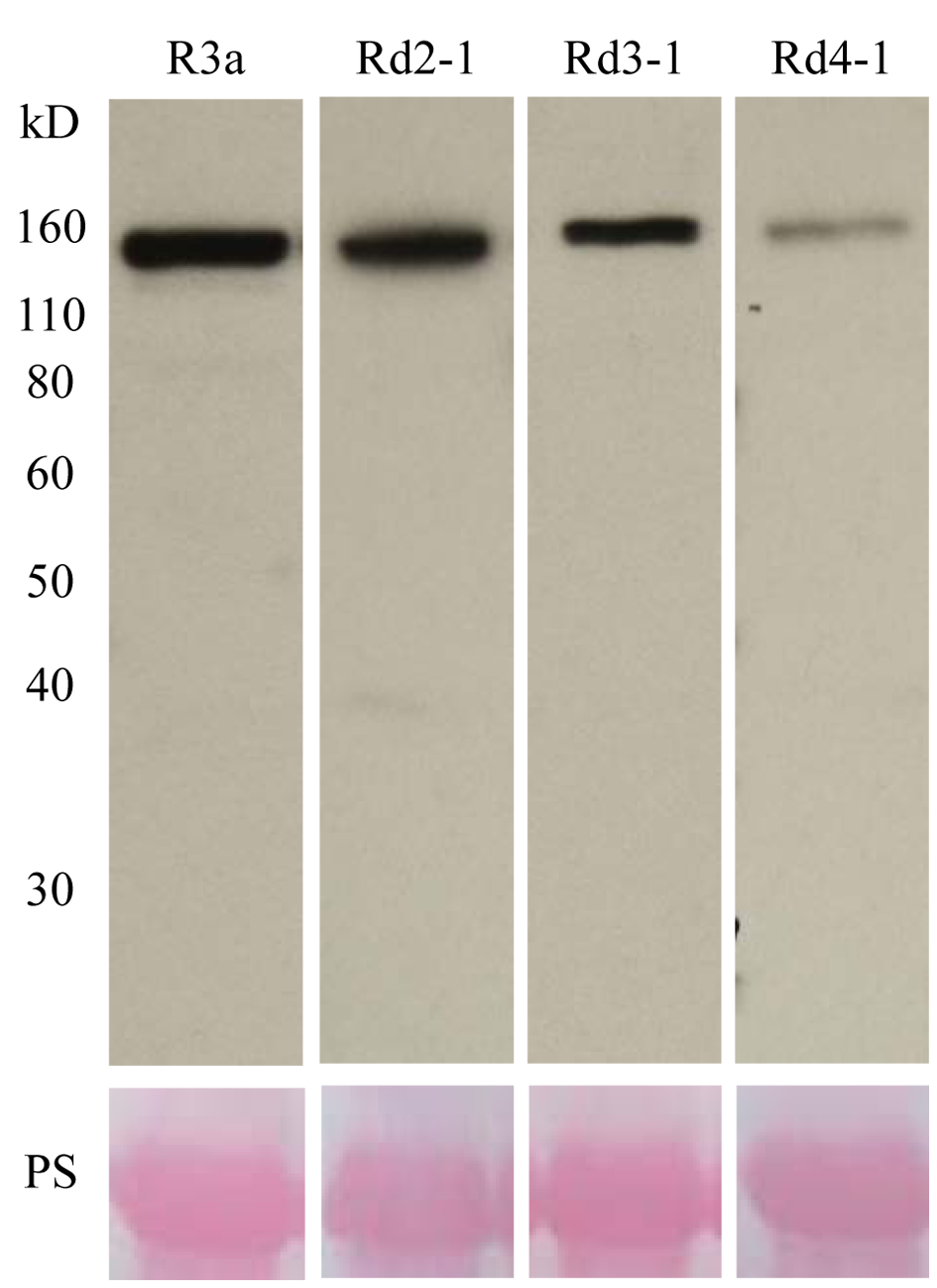

Supplement: Figure S3 — Western blot analysis showing integrity of YFP fusion proteins. Soluble protein extracts were prepared from N. benthamiana leaf tissue two days after infiltration with cultures designed to express YFP fusions to R3a, Rd2-1, Rd3-1 or Rd4-1. The blot was probed with anti-GFP antibodies as described by Engelhardt et al. (2012). Protein sizes are indicated in kilodaltons (kD) and protein loading is shown by Ponceau S (PS) staining. (TIF) [file pone.0110158.s003.tif]

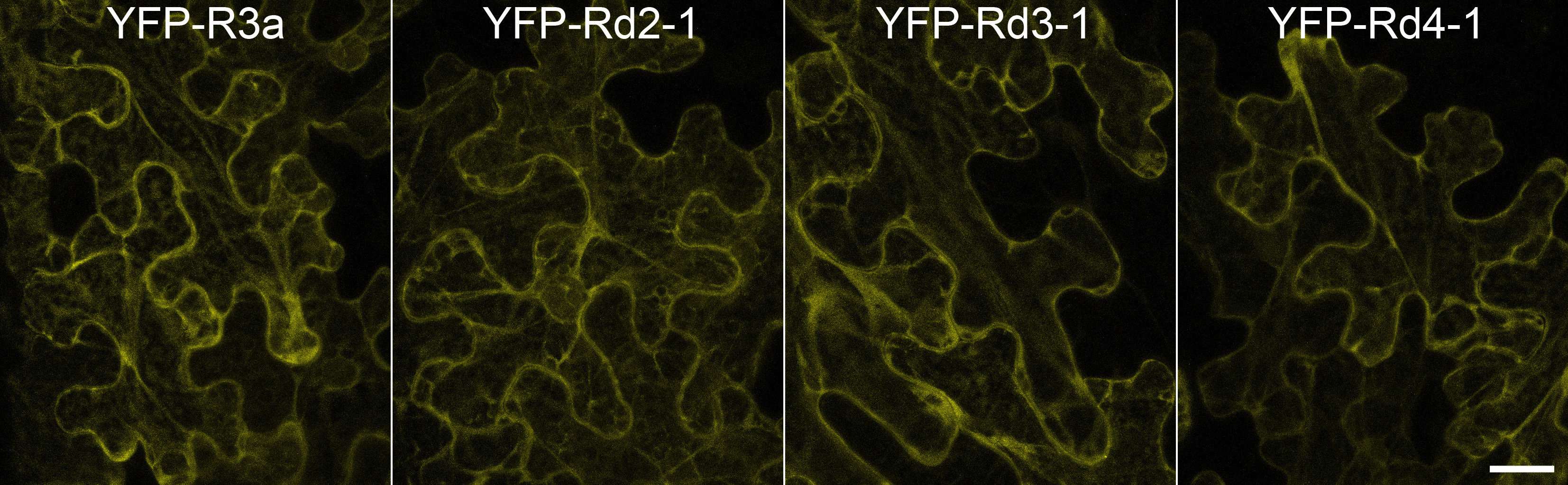

Supplement: Figure S4 — YFP fusions to R3a and the R3a* variants localize to the cytoplasm in the absence of AVR3a. N. benthamiana leaves were infiltrated with cultures designed to express YFP fusions to R3a, Rd2-1, Rd3-1 or Rd4-1. Leaf tissue was examined two days after infiltration under a confocal laser scanning microscope. Representative images are from five independent experiments. Scale bar = 20 µm. (TIF) [file pone.0110158.s004.tif]

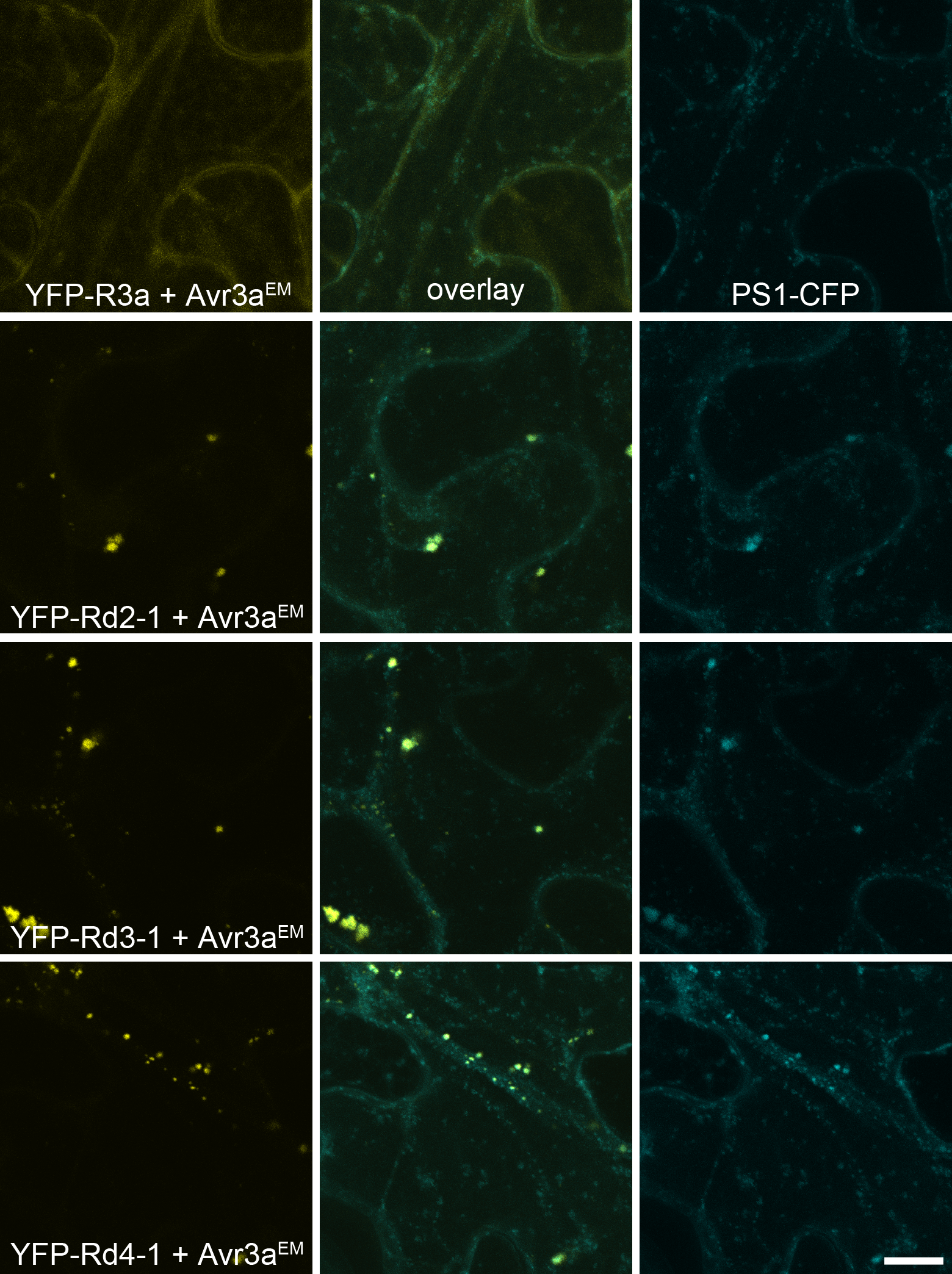

Supplement: Figure S5 — In the presence of AVR3aEM YFP fusions to R3a* variants, but not YFP-R3a, re-localize to vesicles labelled by the prevacuolar compartment marker PS1-CFP. N. benthamiana leaves were infiltrated with mixtures of cultures designed to express PS1-CFP, AVR3aEM and YFP fusions to R3a, Rd2-1, Rd3-1 or Rd4-1. Tissue was examined two days after infiltration under a confocal laser scanning microscope. The left-hand panel shows YFP signal, the right-hand panel CFP signal and the central panel displays the merged signals. Representative images are from three independent experiments. Scale bar = 10 µm. (TIF) [file pone.0110158.s005.tif]

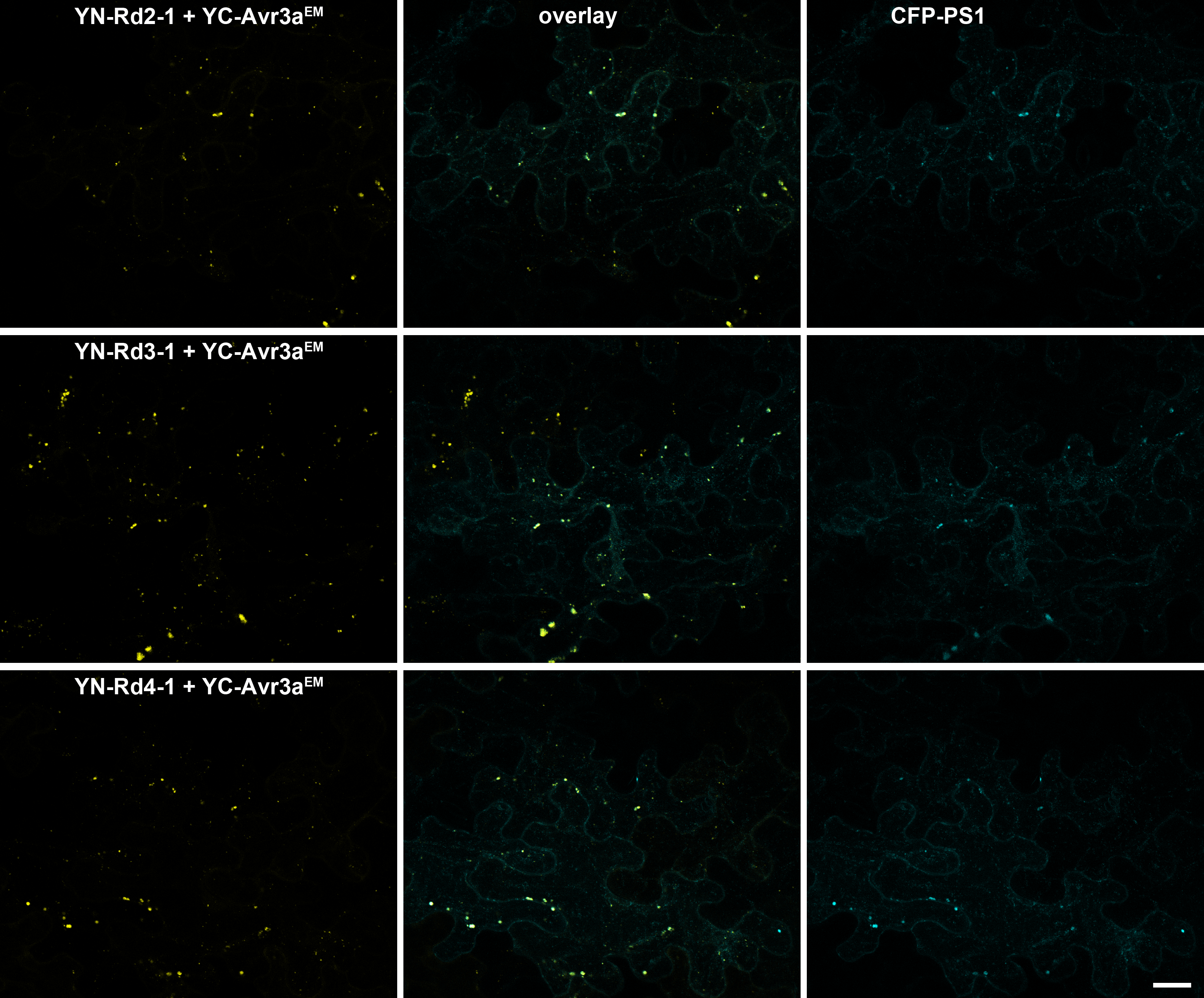

Supplement: Figure S6 — YC-AVR3aEM reconstitutes YFP fluorescence with YN fusions to the R3a* variants at vesicles labelled by the prevacuolar compartment marker PS1-CFP. Generation of the YFP signal indicates that AVR3aEM and the R3a* variants are in close proximity at the vesicles. N. benthamiana leaves were infiltrated with mixtures of cultures designed to express PS1-CFP, YC-AVR3aEM and YN fusions to Rd2-1, Rd3-1 or Rd4-1. Tissue was examined 2 d after infiltration under a confocal laser scanning microscope. Left-hand panel, YFP signal; right-hand panel, CFP signal; central panel, merged signals. Representative images from three experiments. Scale bar = 20 µm. (TIF) [file pone.0110158.s006.tif]
