# Supplementary material for: Detection of the Virulent Form of AVR3a from Phytophthora infestans following Artificial Evolution of Potato Resistance Gene R3a
Source: PLoS One. 2014 Oct 23;9(10):e110158. doi: 10.1371/journal.pone.0110158 (PMC4207746; doi:10.1371/journal.pone.0110158)
Supplement: Table S1 — Primers used in this study. (DOCX) [file pone.0110158.s007.docx]

Primers used in this study.

| Primer name | Primer sequence 5’ -> 3’ |
| --- | --- |
| R3a-5-Asc | TTGGCGCGCCTAAAATGGAGATTGGCTTAGCAGTT |
| R3a-1564-Bam-P | CAAGGATCCCATATGTTGGAACAAAGTC |
| R3a-1564-Bam-M | CAACATATGGGATCCTTGGCTCTCTT |
| R3a-1740W-P | ACTGCCAAGACTARCATCCTTAAGGGC |
| R3a-1740W-M | GCCCTTAAGGATGYTAGTCTTGGCAGT |
| R3a-1841W-P | TGGATATTTCTCRGACAGAKATTAAAAGGTT |
| R3a-1841W-M | AACCTTTTAATMTCTGTCYGAGAAATATCCA |
| R3a-2743W-P | ACCTTGAAGRGAATARAGATATCTGRTTGCCAGAA |
| R3a-2743W-M | TTCTGGCAAYCAGATATCTYTATTCYCTTCAAGGT |
| R3a-3028-M | CAGCCACTTCAGCTTCTTACAGTAGGCAAT |
| R3a-3-Not | TTGCGGCCGCTCACATGCATTCCCTATCGATCT |
