# Supplementary material for: Detection of the Virulent Form of AVR3a from Phytophthora infestans following Artificial Evolution of Potato Resistance Gene R3a
Source: PLoS One. 2014 Oct 23;9(10):e110158. doi: 10.1371/journal.pone.0110158 (PMC4207746; doi:10.1371/journal.pone.0110158)
Supplement: Table S2 — Nucleotide and amino acid changes found in R3a* clones. (DOCX) [file pone.0110158.s008.docx]

Summary of nucleotide and amino acid changes found in recovered R3a variants.

| **Clone** | **Nucleotide change** | **Amino acid** |  | **Clone** | **Nucleotide change** | **Amino acid** |
| --- | --- | --- | --- | --- | --- | --- |
| Rd1-01 | A2758G  A3084G  A3117G | K920E |  | Rd2-01 | T1605C  A1753G  G1860T  A2376G  T3306C | T585A  E620D |
|  |  |  |  |  |  |  |
|  |  |  |  |  |  |  |
| Rd1-02 | A2792G  T3306C  A3435G | Q931R |  |  |  |  |
|  |  |  |  |  |  |  |
|  |  |  |  | Rd2-02 | G1860T  A2768G  A3435G | E620D  D923G |
| Rd1-03 | G1853A  T2005C  A2376G  G3210A | R618Q |  |  |  |  |
|  |  |  |  |  |  |  |
|  |  |  |  | Rd2-03 | G1860T  T3156C  G3210A  T3391C  A3561G | E620D  F1131L |
|  |  |  |  |  |  |  |
| Rd1-04 | G1860T  T2235C  T2671C | E620D  S891P |  |  |  |  |
|  |  |  |  |  |  |  |
|  |  |  |  |  |  |  |
| Rd1-05 | T1698A  A1819G  A3431G | K607E  E1144G |  | Rd2-04 | G1860T  A2752G  T2785C  A3011G  A3046G  T3156C  A3435G  A3792C | E620D  R918G  Q1004R  L1016V  E1264D |
|  |  |  |  |  |  |  |
|  |  |  |  |  |  |  |
| Rd1-06 | A1635G  A2573G  A2752G  T2785C  T3378G | Q858R  R918G |  |  |  |  |
|  |  |  |  |  |  |  |
|  |  |  |  |  |  |  |
|  |  |  |  |  |  |  |
|  |  |  |  |  |  |  |
| Rd1-07 | T1605C  A1753G  A2304T  T2919A  C3198T  C3503T | T585A  S1168L |  | Rd3-01 | G1860T  T2003C  T2235C  A2792G  T3156C  G3210A | E620D  L668P  Q931R |
|  |  |  |  |  |  |  |
|  |  |  |  |  |  |  |
|  |  |  |  |  |  |  |
|  |  |  |  |  |  |  |
|  |  |  |  |  |  |  |
| Rd1-08 | T1870C  A3011G  A3561G  G3637A  T3780C | Q1004R  E1213K |  | Rd3-02 | T1759C  G1860T  A2612T  A2758G  A3117G  T3490C | E620D  E871V  K920E  S1164P |
|  |  |  |  |  |  |  |
|  |  |  |  |  |  |  |
|  |  |  |  |  |  |  |
|  |  |  |  |  |  |  |
| Rd1-09 | T1829C  A2841G  T2922C  T3391C  A3784G | L610P  F1131L  K1262E |  |  |  |  |
|  |  |  |  | Rd3-03 | G1860T  T2235C  T2671C  A2752G  T2785C  A2792G  A3011G  C3097A | E620D  S891P  R918G  Q931R  Q1004R  L1033M |
|  |  |  |  |  |  |  |
|  |  |  |  |  |  |  |
|  |  |  |  |  |  |  |
| Rd1-10 | T2003C  A2091G  A2768G  C3046G  T3156C | L668P  D923G  L1016V |  |  |  |  |
|  |  |  |  |  |  |  |
|  |  |  |  |  |  |  |
|  |  |  |  |  |  |  |
|  |  |  |  | Rd4-01 | G1853A  G1860T  A2758G  A2792G | R618Q  E620D  K920E  Q931R |
| Rd1-11 | A1808C  C3100T  T3490C  A3792C | D603A  H1034Y  S1164P  E1264D |  |  |  |  |
|  |  |  |  |  |  |  |
|  |  |  |  |  |  |  |
|  |  |  |  | Rd4-02 | Rd4-01 +  A1753G | Rd4-01 +  T585A |
|  |  |  |  |  |  |  |
|  |  |  |  | Rd4-03 | Rd4-01 +  C2260A | Rd4-01 +  P887H |
|  |  |  |  |  |  |  |
|  |  |  |  | Rd4-04 | Rd4-01 +  A1753G  G1976T | Rd4-01 +  T585A  R659L |
|  |  |  |  |  |  |  |
|  |  |  |  |  |  |  |
