# Supplementary material for: Detection of the Virulent Form of AVR3a from Phytophthora infestans following Artificial Evolution of Potato Resistance Gene R3a
Source: PLoS One. 2014 Oct 23;9(10):e110158. doi: 10.1371/journal.pone.0110158 (PMC4207746; doi:10.1371/journal.pone.0110158)
Supplement: Table S3 — Mean symptom scores from four nine-day experiments for upper and lower leaves. (DOCX) [file pone.0110158.s009.docx]

Mean disease scores for infiltrated mixtures.

| Mixture | Upper leaf | Lower leaf |
| --- | --- | --- |
| R3a & EM | 0.705 | 0.842 |
| Rd2-1 & EM | 2.152 | 3.245 |
| Rd3-1 & EM | 3.799 | 5.06 |
| Rd4-1 & EM | 4.265 | 5.376 |
| R3a & KI | 4.778 | 5.742 |

Standard error = 0.08749
